# Quality over quantity - rethinking social participation in dementia prevention: results from the AgeWell.de trial

**DOI:** 10.1007/s00127-024-02757-4

**Published:** 2024-09-09

**Authors:** Robert P. Kosilek, Flora Wendel, Isabel Zöllinger, Hanna Lea Knecht, Iris Blotenberg, Solveig Weise, Thomas Fankhänel, Juliane Döhring, Martin Williamson, Melanie Luppa, Andrea E. Zülke, Christian Brettschneider, Birgitt Wiese, Wolfgang Hoffmann, Thomas Frese, Hans-Helmut König, Hanna Kaduszkiewicz, Jochen René Thyrian, Steffi G. Riedel-Heller, Jochen Gensichen

**Affiliations:** 1 Institute of General Practice and Family Medicine, University Hospital, LMU Munich, Munich, Germany; 2 Institute and Clinic for Occupational, Social and Environmental Medicine, University Hospital, LMU Munich, Munich, Germany; 3 Institute for Medical Information Processing, Biometry and Epidemiology (IBE), LMU Munich, Munich, Germany; 4 German Centre for Neurodegenerative Diseases (DZNE), site Rostock/Greifswald, Greifswald, Germany; 5 Institute of General Practice and Family Medicine, Martin-Luther-University Halle-Wittenberg, Halle (Saale), Germany; 6 Institute of General Practice, University of Kiel, Kiel, Germany; 7 Institute of Social Medicine, Occupational Health and Public Health (ISAP), Medical Faculty, University of Leipzig, Leipzig, Germany; 8 Department of Health Economics and Health Service Research, University Medical Centre Hamburg-Eppendorf, Hamburg, Germany; 9 Work Group Medical Statistics and IT-Infrastructure, Institute for General Practice, Hannover Medical School, Hannover, Germany; 10 Institute for Community Medicine, University Medicine Greifswald (UMG), Greifswald, Germany; 11 Faculty V: School of Life Sciences, University of Siegen, Siegen, Germany

**Keywords:** Social participation, Lifestyle intervention, Prevention, Covid-19, Dementia, Randomized controlled trial

## Abstract

**Background:**

Social participation as a protective factor against cognitive decline was one of the targets in the AgeWell.de study, a multi-domain interventional trial in a sample of older adults at increased risk for dementia. This study aimed to examine differential effects of the intervention and other influencing factors on social participation throughout the trial.

**Methods:**

A longitudinal analysis of study data at the primary follow-up after 24 months (*n* = 819) was conducted. The Lubben Social Network Scale (LSNS-6) was used to assess quantitative aspects of social networks, and self-reported social activities were classified using a three-tiered categorical framework to capture qualitative aspects.

**Results:**

A positive effect of the intervention was observed at the qualitative framework level, with an OR of 1.38 [95% CI: 1.05–1.82] for achieving or maintaining higher social participation at follow-up, while no effect could be detected on quantitative social network characteristics. Later phases of the Covid-19 pandemic showed a negative impact on the level of social participation at follow-up with an OR of 0.84 [95% CI: 0.75–0.95].

**Conclusions:**

These findings suggest that by focusing on qualitative aspects of social participation as a component of dementia prevention, future interventions can promote enriched social interactions within established social networks.

**Trial Registration:**

German Clinical Trials Register (DRKS) ID DRKS00013555.

**Supplementary Information:**

The online version contains supplementary material available at 10.1007/s00127-024-02757-4.

## Background

As the global population continues to age with rising life expectancy, understanding what makes healthy aging possible and how to preserve cognitive function as we get older has become one of the top priorities for public health research. The *Lancet* commission on dementia prevention, intervention and care has identified social isolation as one of twelve major modifiable risk factors, based on observational evidence linking it to cognitive decline, while enhanced social participation may provide a protective effect through cognitive stimulation, stress reduction, and promotion of healthy behaviors [1–3]. Consequently, social isolation and participation were targeted within recent trials in dementia prevention, such as the AgeWell.de-trial in Germany, which is based on the Finnish Geriatric Intervention Study to Prevent Cognitive Impairment and Disability (FINGER) multi-component intervention against cognitive decline. AgeWell.de is part of the WW-FINGER Network [4–6], aiming to adapt the successful FINGER-intervention to different regional contexts and healthcare systems. However, the primary analysis of this trial did not detect any effect of the intervention on social participation as a secondary outcome [7]. Since the Covid-19 pandemic has considerably affected social participation, especially among older adults, it needs to be considered as a complicating factor in the assessment of current trials [8]. Consequently, this study explores social participation throughout the AgeWell.de-trial in detail, aiming to elucidate the impact of the intervention, Covid-19 pandemic restrictions, and other contributing factors.

## Methods

### Study design

This is a longitudinal analysis of social participation, which was examined as a secondary outcome within the AgeWell.de trial, a cluster-randomized trial of a primary care multi-domain intervention against cognitive decline conducted in Germany between June 2018 and January 2022. The respective study protocol, baseline characteristics, and main results are described in detail in previous publications [5–7]. Study participants were recruited from general practitioner (GP) offices at five trial sites across Germany, with the GP offices serving as randomization clusters. The target population were community-dwelling adults aged 60–77 years, who were at increased risk for developing dementia based on the “Cardiovascular Risk Factors, Aging, and Dementia” (CAIDE) risk score. The multi-domain intervention was delivered by trained study nurses as three face-to-face and five telephone appointments. It targeted social, cognitive, and physical activity, as well as optimization of nutrition and medication. The social participation intervention consisted of setting and reviewing individual goals for social activities during the visits, and encouraging participants to engage in social activities such as community events or family gatherings using a motivational interviewing technique. Participants in the control group received GP treatment as usual and general health advice.

### Subjects & data

Out of 1,030 participants at baseline, 819 completed the follow-up assessment at 24 months. The variables shown in Table 1 were used for the analyses in this study. The 6-item version of the Lubben Social Network Scale (LSNS-6) [9, 10] and self-reported social activity represented the main outcomes, along with sociodemographic variables, the Montreal Cognitive Assessment (MoCA) and Geriatric Depression Scale (GDS) used as covariates [11–13].

**Table 1:** Baseline characteristics of the study sample

Sample Characteristic	*N* = 819
**Sociodemographic data**	
Gender (male), *n* (%)	386 (47.1%)
Age (years), median (IQR)	69.0 (65.0, 73.0)
Living situation, *n* (%)	
*With partner*	534 (65.2%)
*With relatives*	46 (5.6%)
*Alone*	237 (28.9%)
*Care facility*	2 (0.2%)
Relationship status (Single), *n* (%)	246 (30.0%)
Household members, median (IQR)	2.0 (1.0, 2.0)
Education (CASMIN levels), *n* (%)	
*Low*	181 (22.1%)
*Intermediate*	434 (53.0%)
*High*	204 (24.9%)
Working, *n* (%)	170 (20.8%)
**Cognition and depression assessment**	
Montreal Cognitive Assessment (MoCA) score, median (IQR)	25.0 (23.0, 27.0)
Mild cognitive impairment (MoCA ≤ 25), *n* (%)	455 (55.6%)
Geriatric depression scale (GDS) score, median (IQR)	1.0 (0.0, 2.0)
Depression (GDS ≥ 5), *n* (%)	57 (7.0%)
**Social participation**	
LSNS-6 (total score), median (IQR)	18.0 (14.0, 21.0)
LSNS-6 (family subscale), median (IQR)	10.0 (7.0, 12.0)
LSNS-6 (friends subscale), median (IQR)	8.0 (6.0, 11.0)
Isolation (LSNS-6 < 12 points)	133 (16.2%)
Social involvement, *n* (%)	
*None*	90 (11.0%)
*Low*	384 (46.9%)
*High*	345 (42.1%)

Social participation can be defined as “engagement of individuals in social leisure activities (focusing on activities undertaken with other people), contact with social networks and their satisfaction with this participation” [3]. Social networks play a crucial role in facilitating social participation, which encompasses a broad range of activities, such as group recreational activities, social events, or volunteering [14]. For the purpose of this study, our operational definition of social participation focuses on contact with social networks and engagement in social activities based on the available data and instruments: The LSNS-6 measures the quantitative aspect by assessing the size and depth of social networks, while Levasseur’s taxonomy evaluates the qualitative aspect by categorizing social activities based on their nature and the goals they fulfill, thus reflecting the meaning and depth of social involvement.

The LSNS-6 is a validated and commonly used tool to assess social networks among older adults. It captures the self-reported number of social contacts, as well as support and trust with these connections. The LSNS-6 consists of six questions, one set of three for family and one for friends, respectively: How many relatives/friends do you see or hear from at least once a month? How many relatives/friends do you feel at ease with that you can talk about private matters? How many relatives/friends do you feel close to such that you could call on them for help? The questions can be answered on a 5-point Likert scale as follows: 0 (None), 1 (One), 2 (Two), 3 (Three or four), 4 (Five through eight), 5 (Nine or more). Out of a maximum score of 30 points, a value smaller than 12 points has been defined as a cut-off indicating risk for social isolation [9].

As previously described for the analysis of baseline data [15], we additionally assessed social participation according to the framework described by Levasseur et al. [16], which has also been used in other clinical trials [17, 18]. It is based on the goal of the activity and the interaction with others, with more engagement reflected in higher levels of social involvement. Participants were asked about their social participation based on a list of activities, which consisted of social hobbies and events, as well as engagement in a local church, clubs or other volunteer occupations. These self-reported social activities were classified based on the highest ranking answer as “high involvement” if they had a higher level of engagement with a common goal, “low involvement” if they included regular past-time social interactions, and “no involvement” if participants did not regularly engage in any social activities. For example, social hobbies like restaurant or theater visits represented low involvement, while engagement in organizations or other volunteer occupations represented a high level of involvement.

To account for the Covid-19 pandemic in Germany, an additional questionnaire was used to gather data between January and May 2021, assessing the participants’ attitudes and subjective restrictions due to pandemic containment measures. The questionnaire item on social restrictions was used for adjustments in this analysis. Furthermore, a variable indicating the phase during which a participants’ follow-up exam took place, based on the waves of Covid-19 variants of concern according to the German public health institute (RKI) [19], was introduced. Both variables are summarized in Table 2.

**Table 2:** Subjective restrictions and follow-up timepoints during the Covid-19 pandemic

Sample characteristic	*N* = 819
Subjective social restrictions due to Covid-19 pandemic, n (%)	
*None*	115 (14.0%)
*Slightly restricted*	141 (17.2%)
*Moderately restricted*	159 (19.4%)
*Very restricted*	199 (24.3%)
*Severely restricted*	84 (10.3%)
*N/A*	121 (14.8%)
Timepoint of 24-month follow-up relative to Covid-19 pandemic*, n (%)	
*Phase 2: Summer plateau 2020 (until 27-Sep-2020)*	44 (5.4%)
*Phase 3: 2nd wave (until 28-Feb-2021)*	102 (12.5%)
*Phase 4: 3rd wave (VOC Alpha*,* until 13-Jun-2021)*	129 (15.8%)
*Phase 5: Summer plateau 2021 (until 01-Aug-2021)*	114 (13.9%)
*Phase 6a: 4th wave (VOC Delta*,* summer*,* until 03-Oct-2021)*	227 (27.7%)
*Phase 6b: 4th wave (VOC Delta*,* fall/winter*,* until 26-Dec-2021)*	190 (23.2%)
*Phase 7: 5th wave (VOC Omicron BA.1/BA.2*,* until 29-May-2022)*	13 (1.6%)

*Pandemic phases in Germany based on Tolsdorf et al.,* Epid Bull 2022* [19]; *VOC: variant of concern*

Missing data at the single item level amounted to 1–2% for social participation questionnaires, MoCA and GDS scores at baseline, 2–5% for social participation questionnaires at follow-up, and 15% for the Covid-19 questionnaire item. Missing items were completed using a hot deck imputation procedure conditioned on age, gender, and education [20]. The complete case sensitivity analysis, shown in Table A1, did not reveal any relevant differences between observations with complete or incomplete information for the primary outcomes.

### Statistical analysis

**Fig. 1:**
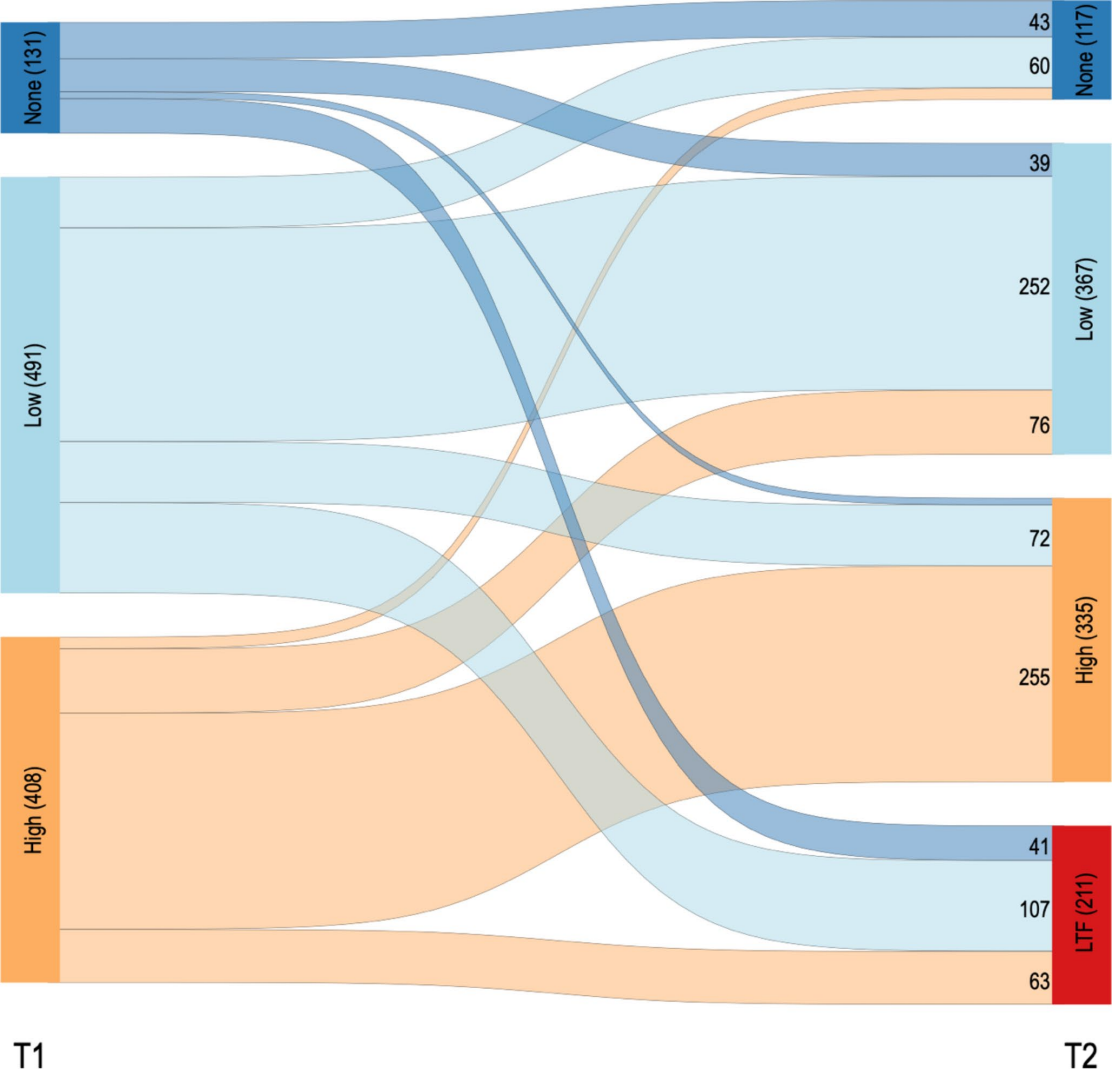
Sankey diagram for the flow of study participants within the social participation framework from baseline (T1) to 24-month follow-up (T2). Social involvement levels based on the framework by Levasseur et al.: None (no regular social contact) - Low involvement (Restaurant visits, sports groups, etc.) - High involvement (Engagement in clubs or volunteer organizations). LTF: Loss to follow-up

Descriptive statistics were used to summarize the baseline characteristics of the study sample. The outcomes of interest for this study were defined as (1) the between-group difference in mean change of the LSNS-6 score at 24-months follow-up, assessed using a two-sample t-test, (2) being at risk for social isolation as a binary outcome, indicated by a LSNS-6 score < 12 points, and (3) the social involvement framework classification as a three-tiered categorical outcome, both compared between groups at 24-months follow-up using Fisher’s exact test. All assessments were done under the intention-to-treat principle as the primary analysis. The mobility of study participants within the social involvement framework from baseline to follow-up was visualized using a Sankey diagram (Fig. 1). [21]

For confirmatory analyses, we then calculated multi-level mixed effects generalized linear regression models, which were adjusted in multiple ways to address potential bias. For the LSNS-6 score, a Gaussian distribution and identity link function were applied, for social isolation, a binomial distribution with logit link was chosen, and an ordered logistic model was applied to the three-tiered framework classification of social involvement, with robust standard errors reported for all models. To account for the cluster-randomized study design, 117 clusters at the GP office level were introduced as random effects. All models were further adjusted for the respective baseline value of the dependent variable, sociodemographic factors (age, gender, education, work, relationship status), as well as depressive symptoms and mild cognitive impairment, all thought to be associated with social participation. A three-tiered factor variable of treatment allocation and protocol adherence, based on goal achievement in the main intervention components [7], was chosen as the main predictor to allow for a per-protocol analysis.

Additionally, the drop-out analysis revealed differences in key variables for those participants lost to follow-up (Appendix, Table A2). To account for potentially resulting informative missingness, a logistic model for study completion, adjusted for significant predictors, was used to derive stabilized inverse probability weights for regression analyses (Appendix, Table A3).

**Fig. 2:**
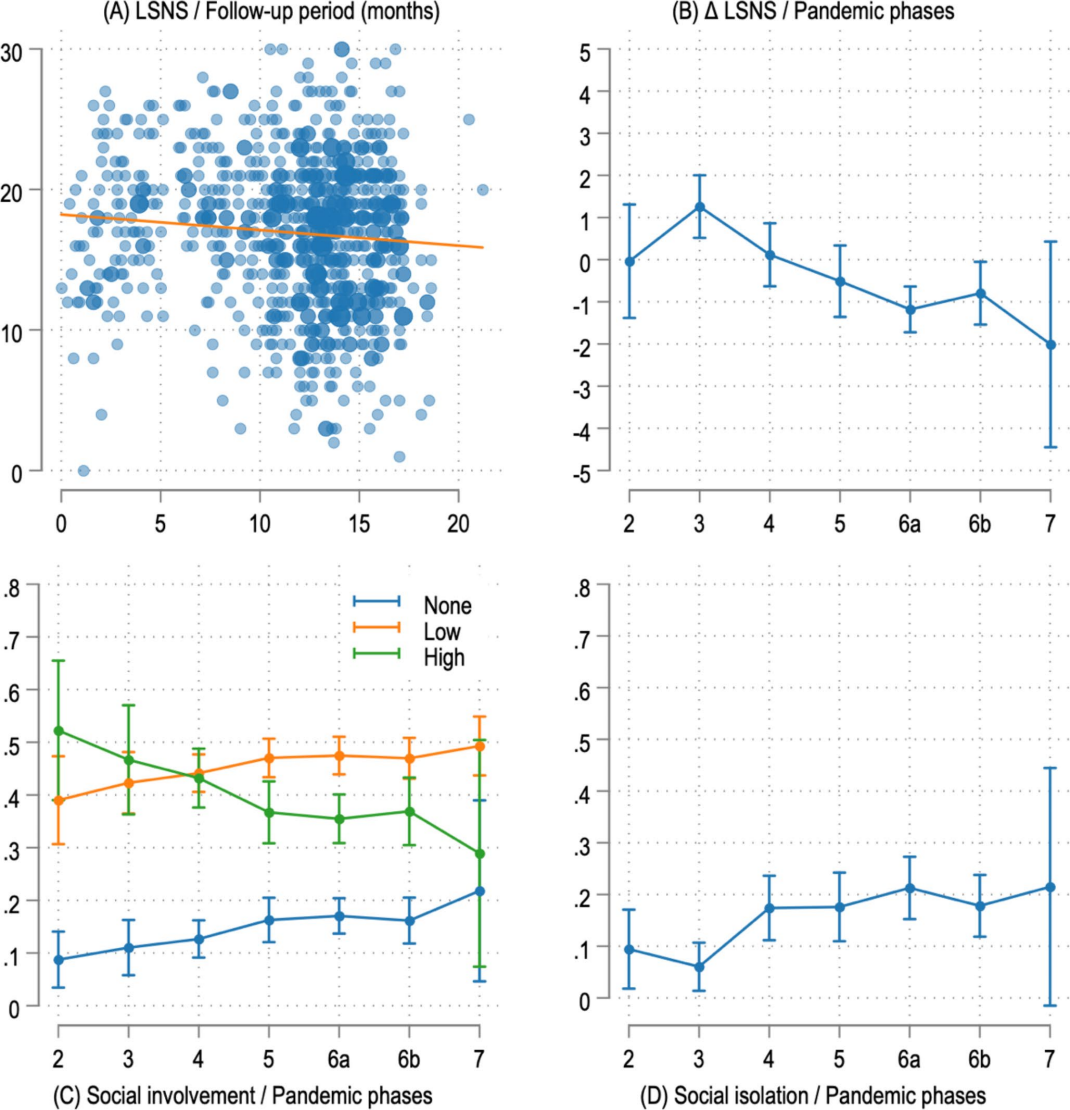
Social participation during the Covid-19 pandemic. Panel **A**: Scatterplot of LSNS-6 scores at 24-month follow-up throughout the follow-up period between July 2020 and April 2022. ß=-0.11 (SE = 0.044, *p* = 0.011). Panels **B** – **D**: Average marginal effects on given outcomes at 24-month follow-up during the respective pandemic phases in Germany between May 2020 and May 2022 [19] as listed in Table 2, obtained from fully adjusted regression models shown in Table 4. Panel **B** shows the change in LSNS-6 scores from baseline, and Panels **C** and **D** show the probability of observing the denoted level of social involvement or risk for social isolation (LSNS-6 < 12) at the respective pandemic phase

Regarding the Covid-19 pandemic in Germany, the association between the pandemic and social participation outcomes at follow-up was visualized, showing a negative trend (Fig. 2, Panel A). Consequently, regression models were adjusted for pandemic effects using the variables shown in Table 2. The fully adjusted models were also used to calculate average marginal effects for the pandemic phases as a factor variable, resulting in Panels B-D shown in Fig. 2.

All statistical analyses were performed using Stata 15.1 (Stata Corp, College Station, TX).

## Results

Table 1 shows baseline characteristics of the study sample. Most participants were living with their partner or relatives. The median MoCA score was 25 points, which translates to a little over half of the participants showing mild cognitive impairment. GDS scores showed a heavily right-skewed distribution due to manifest depression being an exclusion criterion, with only 7% passing the threshold of 5 points indicating at least minor depression. Regarding social participation at baseline, the median LSNS-6 score of 18 points suggested a moderately sized social network for the majority of participants, while about 16% were at risk for social isolation based on a LSNS-6 score < 12 points. Looking at the social involvement framework, 89% were socially active either in simple activities with others or engaged in social organizations. < Table 1 >.

Figure 1 depicts the flow of study participants within the social involvement framework throughout the observation period. Out of the high, low, and no social involvement groups, 63%, 51% and 33% remained in their categories, respectively. Those with no social involvement showed the highest attrition rate with 31% lost to follow-up, compared to 22% and 15% for those with low and high involvement, respectively. Tables A2 and A3 in the Appendix show the drop-out analysis. Study completion was positively associated with higher age, education, cognitive function, and social participation, and negatively associated with depression. < Fig. 1 >.

Measures related to the Covid-19 pandemic are shown in Table 2; Fig. 2. Most follow-up exams took place during the Delta variant wave between August and December 2021. < Table 2 >.

LSNS-6 scores at follow-up showed a negative trend throughout the observation period (Fig. 2, Panels A and B), and later pandemic phases were associated with an increasing probability of observing lower levels of social involvement and higher risk for social isolation (Fig. 2, Panels C and D). < Fig. 2 >.

Table 3 shows comparisons between control and treatment groups for the main outcomes. LSNS-6 scores decreased for both groups by only a small factor, corresponding with the negative trend seen in Fig. 2. There were no meaningful between-group differences in the total score as well as in the subscales. There was also no relevant difference regarding those at risk for social isolation based on a LSNS-6 score < 12 points. However, an intervention effect can be seen in the social involvement framework, where participants in the intervention group showed a higher level of social engagement at follow-up. < Table 3 >.

**Table 3:** Group comparisons for social participation outcomes at 24-month follow-up

Randomization	Control	Treatment	p*
*N*	441	378	
LSNS-6 – Change from baseline, mean (SD)			
*Total score*	-0.4 (4.7)	-0.6 (4.9)	0.62
*Family subscale*	-0.3 (2.7)	-0.3 (2.8)	0.88
*Friends subscale*	-0.1 (3.4)	-0.3 (3.3)	0.56
Isolation (LSNS-6 < 12 points), *n* (%)	77 (17.5%)	57 (15.1%)	0.39
Social involvement level, *n* (%)			0.037
*None*	72 (16.3%)	45 (11.9%)	
*Low*	205 (46.5%)	162 (42.9%)	
*High*	164 (37.2%)	171 (45.2%)	

*Two-sample t-test for continuous variables, Fisher’s exact test for categorical variables

This observed effect is robust to all adjustments. In the regression models in Table 4, the simple model with solely baseline adjustment gives an OR of 1.38 [95% CI: 1.05–1.82] for intervention participants achieving or maintaining higher social involvement at follow-up compared to the control group. The fully adjusted model reveals an even stronger intervention effect with an OR of 1.49 [95% CI: 1.03–2.14] for protocol adherence vs. control group participants. Male gender and single relationship status were positively associated with higher social involvement at follow-up, while depression and later pandemic phases showed a negative association. LSNS-6 scores at follow-up were negatively associated with single relationship status, depression, and the duration of the pandemic. Looking at risk for social isolation based on a LSNS-6 score of less than 12 points, a negative effect of later pandemic phases can be observed with an OR of 1.23 [95% CI: 1.05–1.44], but not for any other covariates. Interestingly, subjective restrictions due to the pandemic did not show any effects on the outcomes. < Table 4 >.

**Table 4:** Regression models for social participation outcomes at 24-month follow-up

Variables	LSNS-6 (Total score)	Isolation (LSNS-6 < 12)	Social involvement level
**Simple model**	(1)	(2)	(3)
Treatment vs. control group	0.10	0.89	1.38*
	[-0.49–0.70]	[0.59–1.33]	[1.05–1.82]
**Fully adjusted model**	(1)	(2)	(3)
Protocol adherence			
*Non-adherent vs. control group*	0.09	1.24	1.07
	[-0.77–0.95]	[0.68–2.25]	[0.58–1.99]
*Adherent vs. control group*	0.09	0.78	1.46*
	[-0.58–0.76]	[0.46–1.33]	[1.02–2.09]
Gender (male)	-0.11	1.02	1.43*
	[-0.71–0.50]	[0.67–1.55]	[1.04–1.98]
Age (years)	-0.01	1.04	1.00
	[-0.07–0.06]	[0.99–1.08]	[0.96–1.03]
Education (CASMIN levels)	0.42	0.78	1.20
	[-0.07–0.92]	[0.59–1.03]	[0.96–1.51]
Relationship status: Single	-0.69*	1.40	1.38*
	[-1.35 - -0.04]	[0.91–2.16]	[1.01–1.88]
Working	0.16	0.79	1.33
	[-0.68–1.00]	[0.46–1.37]	[0.90–1.97]
Mild cognitive impairment (MoCA ≤ 25)	-0.19	0.92	1.01
	[-0.80–0.41]	[0.63–1.34]	[0.74–1.39]
Depression (GDS ≤ 5)	-1.58**	1.54	0.47*
	[-2.72 - -0.44]	[0.74–3.21]	[0.25–0.88]
Subjective social restrictions	0.08	0.92	1.11
	[-0.18–0.34]	[0.78–1.08]	[0.99–1.25]
Phase of Covid-19 pandemic^†^	-0.41**	1.23**	0.84**
	[-0.63 - -0.19]	[1.05–1.44]	[0.75–0.95]

*N* = 819. Linear model with simple coefficients (1), *logistic* (2) *and ordered logistic models* (3) *with odds ratios*, robust 95% CI in brackets. Full mixed-effect models with cluster and drop-out adjustments. Baseline adjustment and constant not shown in table. ** *p* < 0.01, * *p* < 0.05

^†^*Timepoint of follow-up visit relative to pandemic phases in Germany between May 2020 and May 2022* [19]

## Discussion

Our study revealed an interesting contrast between the qualitative and quantitative aspects of social participation in the AgeWell.de trial. While the multi-domain intervention demonstrated a positive effect on social activities based on a qualitative framework, no relevant effects were observed for social network characteristics, quantified by the LSNS-6. Additionally, our results show that the Covid-19 pandemic had a negative impact on social participation, possibly attenuating the effects of the trial on this outcome. We will now discuss these findings in detail.

### Qualitative vs. quantitative aspects of social participation

The first question that needs to be addressed pertains to the LSNS-6 instrument: Why was there only a small negative change on average, and why did we not observe an intervention effect? The overall mean difference in LSNS-6 scores from baseline to follow-up was merely − 0.5 on a 30-point scale. This is most likely due to a compound effect of relative stable social networks among older adults on the one hand and the Covid-19 pandemic on the other: In a 2022 review on the impact of Covid-19 on the psychosocial well-being of older adults, C. Seckman references socioemotional selectivity theory, noting that “older adults favor smaller more intimate relationships as they age and that closeness remains consistent regardless of the frequency of interaction”. while also describing negative effects of the pandemic on social participation [22, 23]. This theory supports the finding that no intervention effect could be seen at the level of social networks, while the estimated impact of the pandemic of -0.4 points on average in our LSNS-6 regression model might almost fully account for the observed change. While the LSNS-6 is an established tool, it is mostly based on the number of connections and the level of trust within those connections, and thus might not be an ideal instrument to capture longitudinal intervention effects under the assumption of smaller, stable social networks in older adults. Future studies with a similar scope and setting might consider exploring other options to capture longitudinal effects on social participation.

The second question pertains to the intervention effects we *did* observe: How can we explain the positive impact on social participation only at the qualitative framework level? In the intervention group, individual social activity goals were encouraged and reviewed during the visits using a motivational interviewing technique, while the control group only received general health advice at the beginning of the study. As a result, a higher level of engagement in social activities was observed for the intervention group using the applied qualitative framework [16]. Participants may have therefore increased their involvement in various social activities without necessarily forming a larger social network due to factors such as the selective nature of social connections in older adults [23]. However, the increase in social involvement may provide benefits such as cognitive stimulation relevant to the aim of the trial, as well as a higher satisfaction with individual social participation.

These results highlight an intriguing contrast between qualitative and quantitative aspects of social participation among older adults. It suggests that focusing on enriching their social lives by meaningful activities within established social networks may hold greater potential for enhancing their social well-being. This distinction calls for a reevaluation of social interventions and survey instruments in older populations, especially since existing instruments are mainly used for rehabilitation research [24].

Regarding the role of other covariates, depression was negatively associated with social participation, which is in line with our previous results and other research [15, 25]. Interestingly, single relationship status was associated with smaller social networks, but higher social involvement, the latter also being true for male gender. This points future trials to subgroups that might either be particularly receptive to social interventions, or conversely require more attention but might also benefit more.

### Unforeseen insights: the role of the Covid-19 pandemic

An interesting and unanticipated finding of our study is the negative impact of the Covid-19 pandemic on social participation among older adults. This unique perspective, resulting from the timing of our data collection coinciding with the onset of the pandemic, allows us to quantify its effects on social networks and participation in our study sample.

Movement restrictions and social distancing measures had made it difficult to conduct research in the outpatient setting, as participants’ willingness and ability to engage in clinical trials may have been affected due to concerns about virus transmission, resulting in recruitment and retention challenges. It is therefore interesting to see that while the pandemic negatively affected social involvement (Fig. 2, Graph 3), the intervention was still associated with achieving or maintaining higher social involvement. This suggests that the intervention mitigated the negative impact of the pandemic to some extent and might even have shown more promising results had it not fallen into the pandemic period.

The Covid-19 pandemic has furthermore drawn the attention of many researchers to the area of social participation as a determinant of health and well-being, especially for older people. There were numerous reviews aiming to assess the impact of containment measures and develop strategies to mitigate the social consequences [8, 22, 26, 27]. Our study contributes to the evolving literature on crisis management in older adults, highlighting the vulnerability of older adults to the social disruptions by quantifying the effects caused by the pandemic, and guiding the development of future interventions.

### Strengths & limitations

One of the strengths of our study lies in its rigorous design, combining data from a randomized controlled trial with a robust and comprehensive statistical analysis. By focusing on the qualitative aspects of social participation, we move beyond traditional metrics of social network size, highlighting future paths for interventions in this area. Additionally, the unexpected insights related to the Covid-19 pandemic add a unique and timely dimension to our research, contributing to the literature on crisis effects in aging populations.

However, our study is not without limitations. As an RCT, it is subject to the limitations inherent in this study design, including potential selection bias, challenges in blinding, and limited applicability beyond the study’s target population. Another potential limitation stems from the fact that the primary outcomes of this analysis were self-reported and might thus be subject to over- or under-reporting. Additionally, while our study provides valuable insights into the impact of the Covid-19 pandemic on social participation, it was not originally designed to investigate pandemic-related effects.

## Conclusions

The findings of our study hold actionable implications for clinical practice and interventions targeting dementia prevention among older adults. By prioritizing qualitative aspects of social participation, interventions can promote enriched social interactions and potentially enhance the social well-being of at-risk older adults. Moreover, the insights related to the Covid-19 pandemic highlight the need to prepare for crises and develop strategies to protect the social well-being of older people. In conclusion, our research advances the understanding of social participation dynamics among older individuals at risk for dementia. By considering both qualitative and quantitative aspects of social engagement and quantifying the unanticipated impact of the Covid-19 pandemic, our study contributes to a more comprehensive approach to dementia prevention and the well-being of older adults.

## Electronic supplementary material

Below is the link to the electronic supplementary material.

Supplementary Material 1: Table A1: Complete case sensitivity analysis. Table A2: Drop-out analysis – Group comparison. Table A3: Drop-out analysis – Logistic model for study completion.

## Data Availability

AgeWell.de data are available for scientific and quality control purposes upon reasonable request based on a data application procedure with the chair of the trial steering committee: Steffi G. Riedel-Heller MD, Institute of Social Medicine, Occupational Health and Public Health, Philipp Rosenthal Str. 55, 04103 Leipzig, steffi.riedel-heller@medizin.uni-leipzig.de.

## References

[1] Livingston G, Huntley J, Sommerlad A, Ames D, Ballard C, Banerjee S et al (2020) Dementia prevention, intervention, and care: 2020 report of the Lancet Commission. Lancet 396(10248):413–446 32738937 10.1016/S0140-6736(20)30367-6 PMC7392084

[2] Penninkilampi R, Casey AN, Singh MF, Brodaty H (2018) The Association between Social Engagement, loneliness, and risk of dementia: a systematic review and Meta-analysis. J Alzheimers Dis 66(4):1619–1633 30452410 10.3233/JAD-180439

[3] Sommerlad A, Kivimaki M, Larson EB, Rohr S, Shirai K, Singh-Manoux A et al (2023) Social participation and risk of developing dementia. Nat Aging 3(5):532–545 37202513 10.1038/s43587-023-00387-0

[4] Ngandu T, Lehtisalo J, Solomon A, Levalahti E, Ahtiluoto S, Antikainen R et al (2015) A 2 year multidomain intervention of diet, exercise, cognitive training, and vascular risk monitoring versus control to prevent cognitive decline in at-risk elderly people (FINGER): a randomised controlled trial. Lancet 385(9984):2255–2263 25771249 10.1016/S0140-6736(15)60461-5

[5] Zülke A, Luck T, Pabst A, Hoffmann W, Thyrian JR, Gensichen J et al (2019) AgeWell.de - study protocol of a pragmatic multi-center cluster-randomized controlled prevention trial against cognitive decline in older primary care patients. BMC Geriatr 19(1):203 31370792 10.1186/s12877-019-1212-1 PMC6670136

[6] Röhr S, Zülke A, Luppa M, Brettschneider C, Weißenborn M, Kühne F et al (2021) Recruitment and baseline characteristics of participants in the AgeWell.de Study-A pragmatic cluster-randomized controlled Lifestyle Trial against Cognitive decline. Int J Environ Res Public Health. ;18(2) 10.3390/ijerph18020408 PMC7825589 33430189

[7] Zülke AE, Pabst A, Luppa M, Roehr S, Seidling H, Oey A et al (2023) A multidomain intervention against cognitive decline in an at-risk-population in Germany: results from the cluster-randomized AgeWell.de trial. Alzheimers Dement 10.1002/alz.13486 PMC10917033 37768074

[8] MacLeod S, Tkatch R, Kraemer S, Fellows A, McGinn M, Schaeffer J et al (2021) COVID-19 era social isolation among older adults. Geriatr (Basel). ;6(2) 10.3390/geriatrics6020052 PMC8162327 34069953

[9] Lubben J, Blozik E, Gillmann G, Iliffe S, von Renteln Kruse W, Beck JC et al (2006) Performance of an abbreviated version of the Lubben Social Network Scale among three European community-dwelling older adult populations. Gerontologist 46(4):503–513 16921004 10.1093/geront/46.4.503

[10] Lubben JE (1988) Assessing social networks among elderly populations. Fam Community Health 11(3):42–52

[11] Nasreddine ZS, Phillips NA, Bedirian V, Charbonneau S, Whitehead V, Collin I et al (2005) The Montreal Cognitive Assessment, MoCA: a brief screening tool for mild cognitive impairment. J Am Geriatr Soc 53(4):695–699 15817019 10.1111/j.1532-5415.2005.53221.x

[12] Sheikh JI, Yesavage JA (1986) Geriatric Depression Scale (GDS): recent evidence and development of a shorter version. Clin Gerontologist: J Aging Mental Health 5(1–2):165–173

[13] Gauggel S, Birkner B (1999) Validität Und Reliabilität Einer Deutschen Version Der Geriatrischen Depressionsskala (GDS). Z für Klinische Psychologie Und Psychother 28(1):18–27

[14] Kelly ME, Duff H, Kelly S, McHugh Power JE, Brennan S, Lawlor BA et al (2017) The impact of social activities, social networks, social support and social relationships on the cognitive functioning of healthy older adults: a systematic review. Syst Reviews 6(1):259 10.1186/s13643-017-0632-2 PMC5735742 29258596

[15] Wendel F, Bauer A, Blotenberg I, Brettschneider C, Buchholz M, Czock D et al (2022) Social Network and Participation in Elderly Primary Care patients in Germany and associations with depressive Symptoms-A cross-sectional analysis from the AgeWell.de Study. J Clin Med 11:19 10.3390/jcm11195940 PMC9572848 36233810

[16] Levasseur M, Richard L, Gauvin L, Raymond E (2010) Inventory and analysis of definitions of social participation found in the aging literature: proposed taxonomy of social activities. Soc Sci Med 71(12):2141–2149 21044812 10.1016/j.socscimed.2010.09.041 PMC3597625

[17] Luster JE, Ratz D, Wei MY (2022) Multimorbidity and Social Participation is moderated by purpose in life and life satisfaction. J Appl Gerontol 41(2):560–570 34225497 10.1177/07334648211027691 PMC8727644

[18] Siren AL, Seppanen M, von Bonsdorff MB (2023) Social Participation considered as meaningful in old age - the perceptions of senior housing residents in Finland. Ageing Int. :1–21 10.1007/s12126-023-09522-z PMC9989560 37359716

[19] Tolksdorf K, Loenenbach A, Buda S (2022) Dritte Aktualisierung Der „Retrospektiven Phaseneinteilung Der COVID-19-Pandemie in Deutschland. Epid Bull 2022 38:3–6

[20] Schonlau M HOTDECKVAR: Stata module for hotdeck imputation. Statistical Software Components S458527, Boston College Department of Economics2018, revised 19 April 2022

[21] Naqvi A (2023) Stata package sankey

[22] Seckman C (2023) The impact of COVID-19 on the psychosocial well-being of older adults: a literature review. J Nurs Scholarsh 55(1):97–111 36218196 10.1111/jnu.12824 PMC9874600

[23] Carstensen LL (2021) Socioemotional selectivity theory: the role of perceived endings in human motivation. Gerontologist 61(8):1188–1196 34718558 10.1093/geront/gnab116 PMC8599276

[24] Schroder D, Heesen G, Heinemann S, Hummers E, Jablonka A, Steffens S et al (2022) Development and validation of a questionnaire to Assess Social Participation of High Risk-Adults in Germany during the COVID-19 pandemic. Front Public Health 10:831087 35558532 10.3389/fpubh.2022.831087 PMC9086897

[25] Beutel ME, Klein EM, Brähler E, Reiner I, Jünger C, Michal M et al (2017) Loneliness in the general population: prevalence, determinants and relations to mental health. BMC Psychiatry 17(1):97 28320380 10.1186/s12888-017-1262-x PMC5359916

[26] Dahlberg L (2021) Loneliness during the COVID-19 pandemic. Aging Ment Health 25(7):1161–1164 33491474 10.1080/13607863.2021.1875195

[27] Smith ML, Steinman LE, Casey EA (2020) Combatting social isolation among older adults in a time of physical distancing: the COVID-19 Social Connectivity Paradox. Front Public Health 8:403 32850605 10.3389/fpubh.2020.00403 PMC7396644

