# Supplementary material for: Quality over quantity - rethinking social participation in dementia prevention: results from the AgeWell.de trial
Source: Soc Psychiatry Psychiatr Epidemiol. 2024 Sep 9;61(6):979–88. doi: 10.1007/s00127-024-02757-4 (PMC13226330; doi:10.1007/s00127-024-02757-4)
Supplement: Supplementary file 1 — Supplementary Material 1: Table A1: Complete case sensitivity analysis. Table A2: Drop-out analysis – Group comparison. Table A3: Drop-out analysis – Logistic model for study completion. [file 127_2024_2757_MOESM1_ESM.pdf]

Appendix

Table A1: Complete case sensitivity analysis for social participation outcomes

Randomization	Control	Treatment	*p**
N	441**	378**	
LSNS-6 – Change from baseline, mean (SD)			
Total score	-0.5 (4.7)	-0.8 (4.9)	0.36
Family subscale	-0.3 (2.7)	-0.4 (2.8)	0.82
Friends subscale	-0.2 (3.3)	-0.4 (3.2)	0.32
Isolation (LSNS-6 <12 points), *n* (%)	79 (18.5%)	62 (16.8%)	0.58
Social involvement level, *n* (%)			0.037
None	68 (15.7%)	43 (11.5%)	
Low	205 (47.5%)	162 (43.4%)	
High	159 (36.8%)	168 (45.0%)	

**Two-sample t-test for continuous variables, Fisher's exact test for categorical variables*
***Missing data for shown variables: <1% at baseline, 2-5% at follow-up*

Table A2: Drop-out analysis – Group comparison

Sample characteristic	Loss to follow-up	Follow-up	*p**
N	211	819	
Age ≥ 70 years, *n* (%)	83 (39.3%)	375 (45.8%)	0.10
Education (CASMIN levels), *n* (%)			<0.001
Low	70 (33.2%)	181 (22.1%)	
Intermediate	112 (53.1%)	434 (53.0%)	
High	29 (13.7%)	204 (24.9%)	
MOCA score, median (IQR)	24.0 (21.0, 26.0)	25.0 (23.0, 27.0)	<0.001
Mild cognitive impairment (MOCA ≤ 25), *n* (%)	150 (71.1%)	455 (55.6%)	<0.001
GDS score, median (IQR)	1.0 (0.0, 3.0)	1.0 (0.0, 2.0)	<0.001
Depression (GDS ≥ 5), *n* (%)	31 (14.7%)	57 (7.0%)	<0.001
LSNS-6 (total score), median (IQR)	16.0 (13.0, 20.0)	18.0 (14.0, 21.0)	0.003
Social involvement, *n* (%)			<0.001
None	41 (19.4%)	90 (11.0%)	
Low	107 (50.7%)	384 (46.9%)	
High	63 (29.9%)	345 (42.1%)	

**Wilcoxon rank-sum test for continuous variables, Fisher's exact test for categorical variables*

Table A3: Drop-out analysis – Logistic model for study completion

Variables	OR	[95% CI]
Age ≥ 70 years	1.44*	[1.05 - 1.99]
Education (CASMIN levels)	1.37*	[1.07 - 1.75]
MOCA score	1.11**	[1.06 - 1.17]
GDS score	0.91*	[0.85 - 0.98]
Social involvement at baseline	1.38**	[1.09 - 1.74]

*N=1,030. Constant omitted. ** p<0.01, * p<0.05.*
